# Population at risk: using areal interpolation and Twitter messages to create population models for burglaries and robberies

**DOI:** 10.1080/15230406.2017.1304243

**Published:** 2017-03-30

**Authors:** Ourania Kounadi, Alina Ristea, Michael Leitner, Chad Langford

**Affiliations:** a Doctoral College GIScience, Department of Geoinformatics-Z_GIS, University of Salzburg, Austria; b Department of Geography and Anthropology, Louisiana State University, Baton Rouge, LA, USA

**Keywords:** Areal interpolation, Twitter, population at risk, spatial crime analysis

## Abstract

Population at risk of crime varies due to the characteristics of a population as well as the crime generator and attractor places where crime is located. This establishes different crime opportunities for different crimes. However, there are very few efforts of modeling structures that derive spatiotemporal population models to allow accurate assessment of population exposure to crime. This study develops population models to depict the spatial distribution of people who have a heightened crime risk for burglaries and robberies. The data used in the study include: Census data as source data for the existing population, Twitter geo-located data, and locations of schools as ancillary data to redistribute the source data more accurately in the space, and finally gridded population and crime data to evaluate the derived population models. To create the models, a density-weighted areal interpolation technique was used that disaggregates the source data in smaller spatial units considering the spatial distribution of the ancillary data. The models were evaluated with validation data that assess the interpolation error and spatial statistics that examine their relationship with the crime types. Our approach derived population models of a finer resolution that can assist in more precise spatial crime analyses and also provide accurate information about crime rates to the public.

## 1. Background: population, crime, time, and place

Population is not randomly distributed in space, but it usually follows a clustering pattern (i.e. has high concentrations in some locations and lower in others). Typically, locations of high concentration have a higher likelihood of crime incidents. However, high concentrations are not easily detected due to peoples’ varying mobility patterns. For example, most people are commuters with varying mobility patterns during work days. These patterns result in spatiotemporal variations of the population and, therefore in spatiotemporal variations of crime occurrences.

The most used population statistic in crime analysis is the residential population. The distribution of population from the census is collected at different spatial scales (e.g. neighborhoods, grid cells, or city level) and contains attributes such as age, gender, occupation, level of education, and others. A variation of the population statistic is to filter and examine specific demographic factors, such as the population of a particular race (H. Zhang, Suresh, & Qiu, 2012), which are assumed to be associated with a higher risk of being a victim of crime. Also, nonresidential mobile population data, termed “ambient,” have been suggested for certain crime types, such as robbery (Zhang et al., 2012), assaults, and violent crime, which do not affect just the resident population but mainly the mobile population. Data that represent the ambient population may reduce the bias that would be produced by the use of the resident population data only. For instance, Stitt, Nichols, and Giacopassi (2003) examined crime occurrences close to casinos and calculated the population at risk by combining census data with tourism data.

Other crime studies used the LandScan global population model to represent the ambient population (Andresen, 2011; Andresen & Jenion, 2008). LandScan has an approximate resolution of 1 km and was modeled by the Oak Ridge National Laboratory (ORNL) using spatial data, imagery analysis, and a multivariable dasymetric modeling approach to disaggregate census counts within administrative boundaries (ORNL, 2016). LandScan data were used to calculate rates of offenses and to identify local spatial autocorrelation using the local Moran’s *I* index (Andresen, 2011; Andresen & Jenion, 2008). A limitation of this dataset is that it estimates a yearly average population and there is no possibility for examining seasonal, weekly, and daily variations of the population.

Another approach is to use social media data as a proxy indicator for the ambient population in spatial crime analysis. Malleson & Andresen (2015a, 2015b, 2016) used Twitter messages to determine if crime hot spots are changing considering different populations (i.e. residential and ambient/mobile). Additionally, the same authors in a more recent publication merged aggregated mobile telephone activity with census and social media data to create an ambient population “collective” dataset (Malleson & Andresen, 2016). However, a drawback of using social media to develop population at risk models is that they underestimate older people living or working in a study area because social media are mostly used by young people (Correa, Hinsley, & De Zuniga, 2010).

The population models of Malleson and Andresen were used in hotspot detection techniques such as the Geographical Analysis Machine (GAM), Getis-Ord (Gi*), and spatial scan statistics to identify spatiotemporal clustering. Other methods that use population information in crime analysis are the thematic mapping of geographic areas as a hot spot method that can be linked with population to calculate crime rates (Chainey, Tompson, & Uhlig, 2008), and prospective predictive techniques such as the Geographically Weighted Regression (GWR) that use population as a parameter estimate of the prediction model (Cahill & Mulligan, 2007).

It is worth pointing out that a more sophisticated population dataset will not be equally accurate when used in spatial statistical techniques of different crime types. That is because there are significant spatiotemporal variations of crime prevalence by crime type. For example, seasonal crime patterns vary by crime type and geography (Andresen & Malleson, 2015). Summer months present a crime increase for some types of crimes (e.g. assault, theft), especially in places where there are a lot of summer activities (e.g. beaches, parks). Also, the decrease and increase of temperature at the hourly level can help to predict street robbery (Tompson & Bowers, 2015).

Furthermore, crime research has looked into the weekly, daily, and hourly variations of crime prevalence and when temporal peaks occur by crime types. For instance, street robberies are most likely to occur during the week during business hours (6 am–5:59 pm) and also during leisure hours (6 pm–1:59 am) (Irvin-Erickson, 2014). Nevertheless, while a specific location is considered a hotspot at night, it may become a coldspot during the day (Caplan & Kennedy, 2011).

On the other hand, robbery prevalence increases at night and on weekends. It is concentrated between 8:00 pm and midnight during the workweek and between 9:00 pm and 4:00 am on weekend nights (Caplan & Kennedy, 2011; Perry, 2013). Regarding assaults, they are also most prominent during weekends (i.e. Friday night to Sunday morning) and are spatially connected with the proximity to bars or nightclubs (Andresen & Malleson, 2013, 2015; Caplan & Kennedy, 2011). Robberies and assaults present temporal similarities and peak periods that are found to be in the early morning hours on weekends and between 00:00 and 03:00 am on Saturdays and Sundays (Ceccato & Uittenbogaard, 2014).

On the other hand, according to Caplan and Kennedy (2011), burglary risk levels seem to be heightened during workday morning and afternoon hours when owners are absent due to their general activities away from home. Other studies that considered acquisitive crimes showed that property crimes occur more often in the afternoons (highest peak is at 5:00 pm) and take place in the city center and regional commercial centers (Uittenbogaard & Ceccato, 2012), while thefts are decreasing during the weekend (Saturday and Sunday) (Andresen & Malleson, 2015).

Moreover, spatial variations of crime prevalence are studied through hot spot analysis. Similar to the temporal variations, crime types show different patterns in space. The spatial diversity of crime is a core component of crime pattern theory as it considers crime attractors and generators as places that result in certain areas being vulnerable to crime (Brantingham & Brantingham, 1981). For example, for the cities of Vancouver and Ottawa, it was found that while thefts are concentrated in the central business district and also in shopping areas across the city, most burglaries occur in residential areas (Andresen & Linning, 2012). In Stockholm, violent crime clusters are concentrated in specific locations in the suburbs, mostly in the weekends (Uittenbogaard & Ceccato, 2012). Locations of alcohol outlets show positive spatial correlation with aggravated assaults (Snowden & Pridemore, 2013). Locations of high drug activity are also positively correlated with aggravated assault as well as with other violent crimes (Snowden & Pridemore, 2013). Among others, these examples identify specific environmental characteristics for each crime type, which support the idea of using non-aggregated crime types in spatial analysis.

## 2. Research gap and the current study

The objective of this study is to use social media information to produce population at risk models for crime (from hereafter “Pop.CR models”). Population at risk is a terminology used mainly in epidemiology and health geography (Ashford, Desjeux, & deRaadt, 1992; Criqui, Denenberg, Langer, & Fronek, 1997; Hay, Guerra, Tatem, Noor, & Snow, 2004). A general definition describes a group of people who are exposed to danger or harm more than the general population (TheLawDictionary, n.d.). Considering crime, population at risk is a model of the general population that represents people who are more likely to become victims of crime. In the previous section, we discussed how population is the core input in spatial crime analysis. Up to now, when spatial analytical methods require population information, most studies use the residential population and few recent studies have used ambient population (Malleson & Andresen, 2015b, 2016). Residential population is inappropriate for crime types that involve mobile population (e.g. street robbery) and although current efforts at ambient population modeling offer improvements for such crime types, these models are still too generic to capture particular characteristics of the vulnerable population (e.g. temporal aspects). To create more precise population models that reflect the distribution of a population when they are at a heightened victimization risk, additional factors can be considered such as place, time, individuals’ traits, and so on. Such versatile information is available with data from location-based social networks. In this study, we use the Twitter application and we process the temporal information of geo-located tweets to create spatial distributions for time slots when crime is at its peak.

Geo-located tweets alone can reveal the spatial distribution of the ambient population (Malleson & Andresen, 2015a). Consequently, geo-located tweets that are processed by time intervals can reveal the spatial distribution of a population at risk of crime. However, a geo-located Twitter dataset cannot substitute for a population model due to the inconsistencies between the number of tweets and population. Furthermore, because the amount of tweets per user may vary substantially (Li & Goodchild, 2013), a tweets’ distribution is likely to be biased according to the locations of the “heavy” users. Thus, Twitter data should be preprocessed and used in combination with general population data. In particular, spatial population datasets can be used to control and retain population counts within areas while Twitter data designate their distribution.

We use density weighted areal interpolation to combine population with Twitter data. Areal interpolation is a common method in geographic studies and its main applications are isopleth mapping and transforming data from one set of boundaries (i.e. source zones) to another (i.e. target zones) (Lam, 1983). The interpolation problem has led to the development of several algorithms and application studies. One of the early and most common applications is the disaggregation of census data into a finer grid cell size resolution (Martin, 1989; Mennis, 2003). Apart from creating population surfaces, areal interpolation has also been used for socioeconomic data. Tested interpolated variables include Black population, Hispanic population, number of children, number of households, and values of housing units (Eicher & Brewer, 2001; Mennis & Hultgren, 2006).

Interpolation algorithms involve simple and more advanced methods that employ additional ancillary data (Hawley & Moellering, 2005). The area-weighting method is the simplest approach to interpolate values of a variable as a weighting function of the density values of all source zones’ intersecting with a target zone by assuming homogeneity in values within source zones (Lam, 1983). A similar assumption that the density within each source zone is a constant is also the first part of the Tobler’s (1979) pycnophylactic method. However to counterbalance for heterogeneity in space, the pycnophylactic method considers as a next step the density of the target zones’ neighbors using a smoothing function in each cell. A third popular method that does not include ancillary data is the centroid-based method proposed by Martin (1989) that applies a distance-based decay function from the centroid of each source zone to allocate the value of the variable into each target zone. A major disadvantage of this method is that it does not preserve the total value of each source zone (i.e. volume preserving characteristic). Moving into the commonly known intelligent approaches that employ ancillary data, the dasymetric approach by Fisher and Langford (1995) used 2-D binary land use data. The dasymetric approach discards the homogeneity of source zones but employs it within the control zones (in this example the land use types). A rescaling operator was added later to preserve the volume of each source zone (Langford, 2006). Next, overlaid network algorithms are a group of simple and more complicated methods that employ 1-D road network data (Xie, 1995). Last, the area-to-point interpolation by Kyriakidis (2004) originates from classic geostatistical point interpolations.

In this research, Twitter data act as ancillary data similar to most recent approaches in areal interpolation that involve the use of point data (0-dimension) (Bakillah, Liang, Mobasheri, Jokar Arsanjani, & Zipf, 2014; Lin & Cromley, 2015; Zhang & Qiu, 2011). Due to their 0-dimension (i.e. locations), Twitter data have an additional advantage. Original population data can be disaggregated into zonal systems of finer resolutions. Given the fact that there is spatial heterogeneity in crime rates at a “micro” level (i.e. street to street variation) (Groff, Weisburd, & Yang, 2010), crime trends need to be examined at large scales (i.e. “micro places”). This means that fine resolution risk models produced from point level ancillary data will offer more accurate results than risk models of a coarser resolution, when used in spatial crime analysis applications.

Lastly, to the best of the authors’ knowledge, there has been only one attempt to use social media data for areal interpolation of population. Lin and Cromley (2015) used geo-located tweets along with land cover, roads, and parcels to disaggregate population values from census tracts to block groups in Hartford County, CT. Results showed that adding Twitter data decreases interpolation errors but performs worse in regarding the errors compared to other ancillary data when used as a single layer of information in the interpolation process. Due to the involvement of social media in the areal interpolation process is still a novel endeavor, this study offers additional findings and allows the comparison of results. Nevertheless, it is important to keep in mind that there is no direct analogy between the study of Lin and Cromley (2015) and this study, because the modeling of population at risk for crime is not always a pure residential population model as it also involves the tuning of the temporal information of tweets.

## 3. Modeling population at risk

### 3.1. Density weighted areal interpolation technique

**Figure 1.**
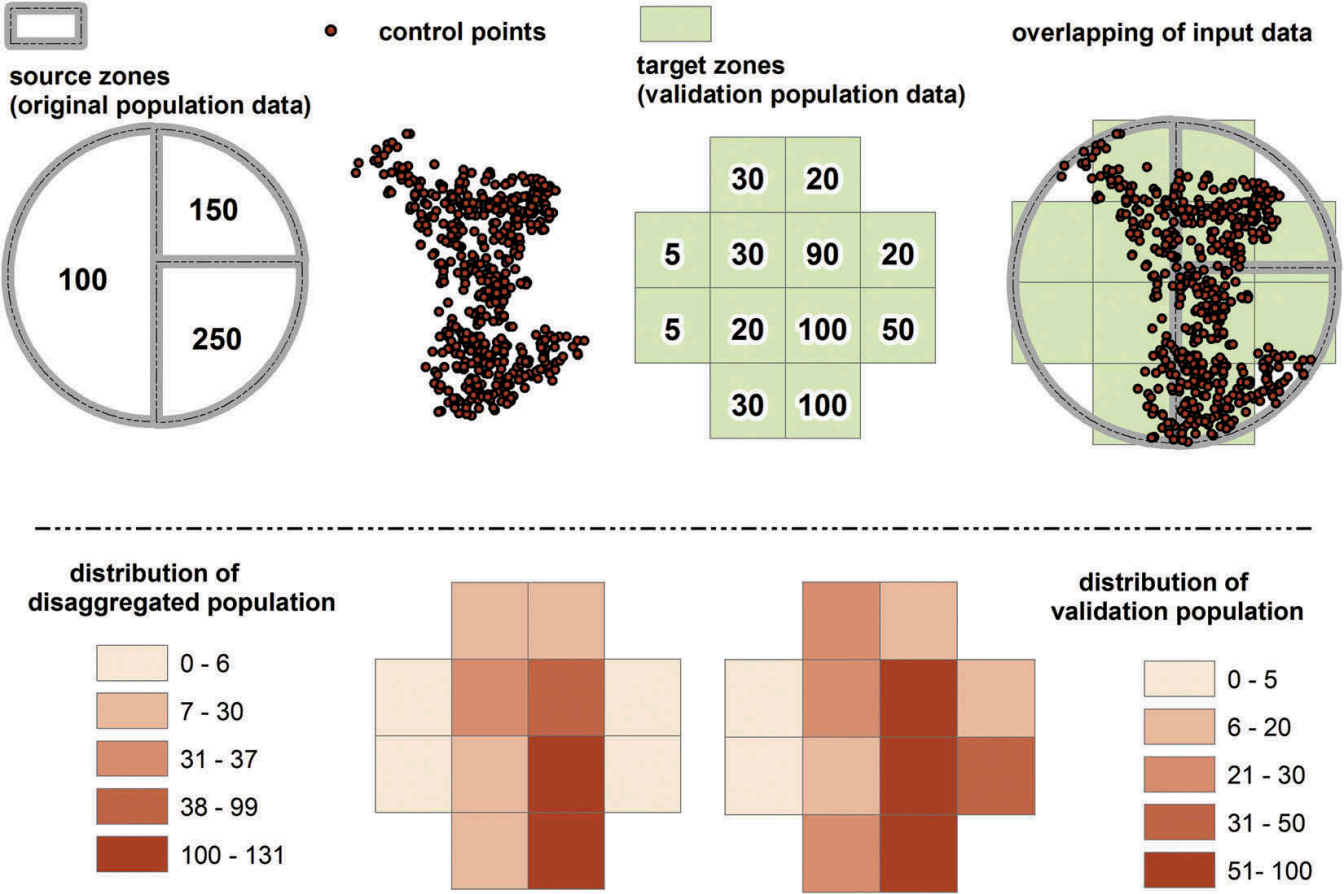
An example of the density weighted areal interpolation technique depicting the three input data (i.e. source zones, control points, and target zones), the output disaggregated model (i.e. distribution of disaggregated population), and if it is available, the distribution of the validation data (i.e. distribution of validation population).

To create Pop.CR models, we used a disaggregation technique as shown in Figure 1. The upper part of the figure shows the input data, namely source zones, control points, and target zones. Source zones are original units (e.g. administrative regions) for which population data are available. The second input data are control points, which represent the spatial distribution of people at crime risk and are used to disaggregate the original units and reallocate their distribution based on the distribution of the risk. The new distribution is transferred to another zonal system, the target zones, which are preferably of a finer resolution. In this study, target zones are grid cells containing population information per cell (validation data), thus allowing the validation of the disaggregating process. Last, the bottom part of the figure shows the density distributions of the disaggregated population and the validation population.

Our technique is based on an areal interpolation technique that was developed by Zhang and Qiu (2011) and uses points as ancillary data. More specifically, the authors used schools as control points to disaggregate residential population from census tracts to ZIP codes in Collin County, TX. In the original method, the source zones are disaggregated to cells and then transferred to the target zones. In our version, the cells are the target zones. Also, the “point-based intelligent approach” uses a function that is based on the distance to the control points, though here we consider the density of the control points. Distance to control points is a reasonable choice when these points represent locations around which the density of the population is higher (in other words hotspot locations). However, our control points (i.e. Twitter data) better represent a density surface of the population. Thus, the distance decay function would result in an inaccurate disaggregation for many cells. For example, a cell that has one control point would get a similar population value compared to a cell that has many control points. The formulas that were used to perform the disaggregation and calculate the values of each target zone are shown below in Equations (1–2).

Our approach is applicable when target zones are smaller units than source zones. Therefore, some target zones can be fully contained inside source zones, while other target zones intersect with source zones, or target zones are only contained by source zones. For example, source zones can be municipalities and target zones grid cells of 0.5 km^2^ resolution. Thus, some cells will be contained within a municipality while others will intersect its boundary. Another example is using districts as source zones and postcodes as target zones. Postcodes typically lie within a district and are aligned with its boundary. At first, each target zone is assigned to one source zone using a *point-in-polygon* operation, where the point is the center of the target zone and the polygon is the source zone that contains that point. The population value for each target zone (*V_si_*) is a function of the ratio of the population value and the sum of weights of the source zone *s*, and the weight of the target zone within zone *s* (Equation 1). The weight (*W_si_*) is calculated using a density function that assigns weights proportionally to the control points’ density in each target zone (Equation 2). The density-weighted areal interpolation technique has been automated with a Python code for the ArcGIS 10.0 program. The code and user instructions are available for free from the corresponding author upon request.

(1) $$V_{si}\;=\;\frac{V_{s}}{\sum_{i=1}^{N_{s}}W_{si}}W_{si}$$

*V_si_* is the value for target zone *i* within source *s* (*number of people*), *V_s_* is the value of source zone *s* (*number of people*), *N_s_* is the number of target zones within source zone *s*, and *W_si_* is the weight of target zone *i* within source zone *s*.

(2) $$W_{si}={\left(\frac{c_{si}}{c_{s}}\right)}^{q}$$

*c_si_* is the number of control points in target zone *i*, *c_s_* is the number of control points within all target zones in source zone *s*, and *q* is the power parameter that controls the degree of influence.

It is worth pointing out that Bakillah et al. (2014) proposed a building level disaggregation approach in an application with points-of-interest (POIs) from OpenStreetMap (OSM) that are associated with a higher density of population. Their approach merged the “point-based intelligent approach” with other existing methods. Similar to Twitter data, the population correlated POIs, due to their volume, resemble a density surface of the population. To reduce the set of POIs and use them as control points in the distance decay function, a quadtree procedure was applied. In a similar effort, we extracted the central features from hotspot areas (i.e. spatial clusters using the nearest neighbor hierarchical clustering technique (Everitt, 1974)) and used these features as control points in a distance decay function as in Zhang and Qiu (2011). The validation that is presented in Section 3.4 was initially performed for both density-weighted and the distance decay functions. The density-weighted function yielded better results and thus only models from this function are presented in the paper.

### 3.2. Analytical strategy

The study area is in Vienna, Austria, where we developed Pop.CR models for two crime types: residential burglary (i.e. burglaries in houses or apartments) and robbery of a cell phone, purse, or bag. Control points varied for each crime type and were created by two different geo-located tweets’ distributions in the study area. For residential burglaries, a Pop.CR model was created, named as “Pop.CR1” by control points, named as “control points 1” that portrayed the distribution of the residential population (i.e. locations of people from Monday to Friday between 2:00 am and 6:00 am – workday sleeping hours). On the other hand, for robberies, we captured the distribution of the population at high crime temporal peaks by analyzing their weekly patterns in the study area. These patterns were identified by analyzing original crime data, which are described in Section 3.3. Hence, a second Pop.CR model was created, named as “Pop.CR2” by control points, named as “control points 2” that portrayed the distribution of the weekend night ambient population (i.e. locations of people from Friday at 4:00 pm to Saturday at 5:00 am, and from Saturday at 4:00 pm to Sunday at 5:00 am).

**Figure 2.**
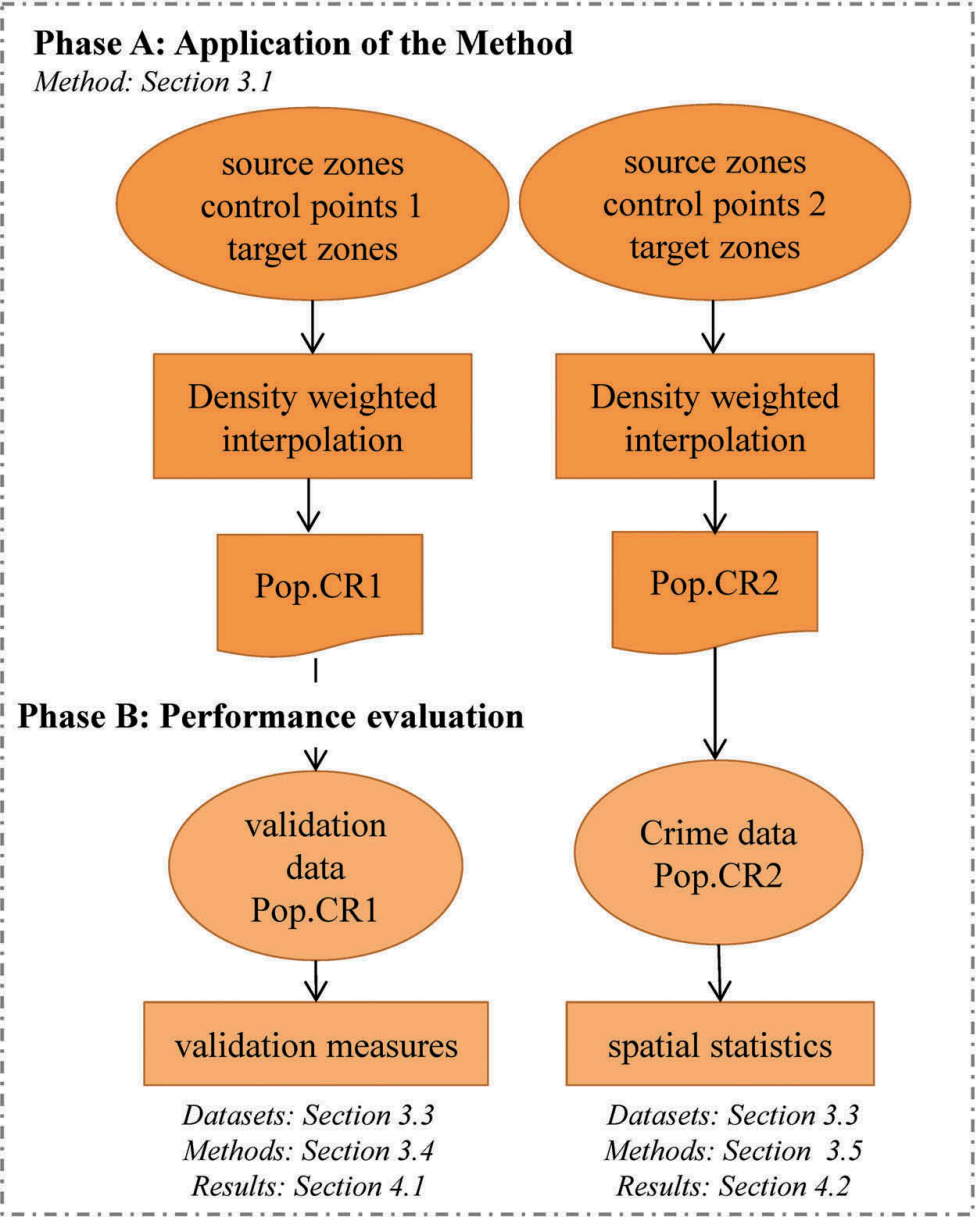
Flowchart of the analytical strategy.

The analytical strategy is presented in Figure 2 and consists of two phases: *phase A* – application of the method, and *phase B* – performance evaluation. In *phase A*, the density weighted interpolation was applied to the input data and the models were created. The input data include source zone data of a coarse resolution, two sets of control points (one for each Pop.CR model), and target zones of a finer resolution. Details of the input data are described in Section 3.3. In *phase B*, the models were evaluated using either validation measures (methods described in Section 3.4 – results shown in Section 4.1) or spatial statistics (methods described in Section 3.5 – results shown in Section 4.2). The validation measures were used only in the first model for which validation population data were available. Population models usually represent residential populations and alternatively ambient populations (Andresen, 2011; ORNL, 2016; Sutton, Elvidge, & Obremski, 2003). Pop.CR2 was evaluated using spatial statistics that examine the spatial relationship of the population at risk and the crime type in question because official  population data with the characteristics of this model currently do not exist. Hence, the original crime data were employed for this part of the analysis, as well.

### 3.3. Datasets and pre-processing

The interpolation method was applied and evaluated for the city of Vienna, Austria. Four types of datasets were used that include the input data (i.e. source zones, control points, and target zones) and crime data (i.e. robberies) in the study area.

#### 3.3.1. Source zones

Source zones are administrative regions with population data per region for 2011. Two types of regions are used: (a) “Bezirk” (i.e. Austrian division equivalent to districts or boroughs), which includes 23 areas of an average size of 18 km^2^, and (b) the entire city of Vienna that has an area of 414.66 km^2^. The population data were obtained by the official statistical service in Austria (StatistikAustria, 2016). The data were not collected by questionnaires to the citizens (traditional census) but directly retrieved from administrative registers. The total population of Vienna in 2011 was 1,714,227 and the population among the Bezirk areas ranged from 16,374 to 177,989 people with a standard deviation of 42,022 people. Bezirk areas are used for the Pop.CR1 model. However, the Pop.CR2 model involves mobile populations for particular time intervals. If Bezirk areas were to be used as source zones for this model, it would imply that people living in these districts move only within the limits of their districts. We assume that Viennese residents may spend their working or leisure time at any location within the city and therefore, we used the entire city as the single-source zone for the second model.

#### 3.3.2. Control points – Twitter data

Twitter data were obtained via harvesting the geo-located messages by streaming the Twitter API for posts in 2012. The administrative boundary of Vienna city was considered the bounding box for the query extracting the tweets. The geo-located dataset contains 303,613 tweets sent by 16,209 users and was further processed so as to extract tweets within temporal intervals that were described in Section 3.2. In total, 7140 tweets were extracted for time intervals that were used to define the residential population (used in PopCR1) and 39,364 tweets for the temporal peaks of robberies (used in PopCR2), respectively. The amount of tweets per user varies substantially (i.e. 1–1050). To counterbalance the tweets discrepancies and restrict the impact that “heavy” users may have in our models, only one location per user was selected. According to Kounadi et al. (Kounadi, Lampoltshammer, Groff, Sitko, & Leitner, 2015), the spatial distribution of tweets by user in the city of London, UK, was found to be highly clustered for most of the users. We tested a sample of 50 randomly selected users and found similar patterns. The average first-order nearest neighbor index (NNI) of locations of the tweets by user was 0.23 (*p* value ≤ 0.001). The results ranged from extremely clustered (i.e. NNI = 0.08) to clustered (i.e. NNI = 0.6) point patterns. Therefore, we concluded that the spatial median is an appropriate centrographic statistic to estimate the location around which a user tweets the most (i.e. a representative location for each user). The spatial median is defined here as the intersection between the median of the *X* coordinates and the median of the *Y* coordinates (Levine, 2013). For each user, we ordered the *X* coordinates and the *Y* coordinates and chose the median of the *X* and the median of the *Y* coordinates. In the case when one user has just one location, we kept these coordinates. The resulting control points set for Pop.CR1 consist of 1354 locations and the set for Pop.CR2 consists of 5445 locations, respectively.

#### 3.3.3. Target zones – validation data

As already mentioned, this study uses target zones for which population data already exist in order to directly validate the results of the interpolation method. In particular, we used the “GEOSTAT 2011” population grid that is produced by Eurostat in cooperation with the European Forum for GeoStatistics (Eurostat, 2016). The grid has a resolution of 1 km^2^ and contains population data for 43 European countries. Regarding Austria, population is obtained from buildings and dwellings registered in 2011. It is estimated that 99.99% of all census buildings were georeferenced and have a positional accuracy of 0.01 m. Also, no disclosure control or confidentiality treatment was applied to the population of each grid cell. For our analysis, Austrian grid cells were selected that intersect the City of Vienna. The cells at the edge of the city extend beyond its periphery and thus, all intersecting cells represent a greater population than the population of the Bezirk areas (i.e. 1,740,953 people in 406 grid cells). The differences in population values between target zones and source zones can affect the validation results. To compensate the difference, only cells having their centers within the study area were selected. The final population grid consists of 352 grid cells and has a total population of 1,707,800 people. The population for individual grid cells ranges from 1 to 28,423.

#### 3.3.4. Crime data – evaluation data

Crime data were provided by the Criminal Intelligence Service Austria (Federal Criminal Police Office) that stores all reported Austrian crime incidents in a database called Security Monitor. The database contains data since 2004 for internal investigation and analysis purposes. For the geocoding of incidents, an Austrian address register database is used. The database contains unique addresses for each building shown on the cadaster and the captured coordinates of crime incidents represent a location inside the footprint of the building. Thus, the positional accuracy of the crime dataset for crimes that can be readily associated with an address is considered fairly high with an estimated range of a few meters. Also, several attribute characteristics are collected and stored for each crime incident. For this study, the agency provided us with the location and temporal information in GIS layers. The crime dataset consists of robberies of a cellphone and robberies of a purse or a bag that were merged together and referred to as robberies in the remainder of this paper (in total 854 incidents). All incidents occurred in Vienna during 2011.

### 3.4. Validation measures

The Pop.CR1 model was evaluated with four error measures that have been used traditionally in the field of areal interpolation. The measures compare the interpolated values (i.e. disaggregated population values) with the actual values in the target zones (i.e. validation population values) and are shown in Table 1.

**Table 1.** Error measures of areal interpolation for evaluation purposes.

Name	Abbreviation	Formula	References
Root mean square error	RMSE	(3) $RMSE\;=\;\sqrt{\frac{\sum_{i\;=\;1}^{N_{i}}{\left(D_{i}\;-\;V_{i}\right)}^{2}}{N_{i}}}$	Bakillah et al. (2014), Zhang and Qiu (2011), Fisher and Langford (1995), Yuan, Smith, and Limp (1997)
Mean absolute error	MAE	(4) $MAE\;=\;\overset{N_{i}}{\underset{i=1}{\sum }}\frac{\left|D_{i}\;-\;V_{i}\right|}{N_{i}}$	Zhang and Qiu (2011), Langford (2006)
Coefficient of determination of linear regression	*R*^2^	(5) $R^{2}=\frac{explained\;variation}{total\;variation}$	Yuan et al. (1997), Bakillah et al. (2014)
Pearson’s correlation coefficient	*r*	(6) $r=\frac{\sum_{i=1}^{N_{i}}\left(V_{i}-\bar{V}\right)\left(D_{i}-\bar{D}\right)}{\sqrt{\sum_{i=1}^{N_{i}}{\left(V_{i}-\bar{V}\right)}^{2}\sqrt{{\left(D_{i}-\bar{D}\right)}^{2}}}}$	Bakillah et al. (2014)

where *V_i_, D_i_*, are, respectively, the validation population value and the disaggregated population value for each target zone. *N_i_* is the number of target zones.

### 3.5. Spatial statistics

Pop.CR2 has the temporal characteristics of a heightened victimization risk for robberies. Therefore, the spatial distribution should be somehow correlated to the spatial distribution of the incidents themselves. Our assumption is that the spatial correlation between the model and its crimes will be higher than if a generic population model was used instead. To test our assumption, we use the Pop.CR1 model, which represents the distribution of the residential population, examine its relationship with the actual locations of robberies, and expect it to be weaker than the relationship between the Pop.CR2 and robberies.

The relationship between distributions of the population models and crime incidents can be calculated using the Pearson correlation coefficient (i.e. Equation 6). However, we employ spatial statistics because global estimates can mislead interpretations or hide local relationships. Recent research shows that variables that appear to be independent using a global correlation coefficient can be significantly locally correlated (Kalogirou, 2012, 2013). Two spatial statistical methods were used to examine the abovementioned relationship, namely, the local Pearson correlation coefficient (Lr) (Kalogirou, 2012; Wheeler & Tiefelsdorf, 2005) (Equation 7) and the GWR (Fotheringham, Brunsdon, & Charlton, 2003) (Equation 8).

(7) $$Lr_{i}=\frac{\sum_{j=1}^{K}\left(C_{j}-\bar{C_{i}}\right)\left(D_{j}-\bar{D_{i}}\right)}{\sqrt{\sum_{j=1}^{K}{\left(C_{j}-\bar{C_{i}}\right)}^{2}}\sqrt{\sum_{j=1}^{K}{\left(D_{j}-\bar{D_{i}}\right)}^{2}}}$$

$Lr_{i}$ is calculated for every point *i*, where *i* is the center point of each target zone, *k* is the number of nearest center points of target zones around this point, $\bar{C_{i}}$ and $\bar{D_{i}}$ are the mean values of the *k* nearest neighbors for robberies and the disaggregated population, respectively.

(8) $$Y_{i}\;=\;\beta_{0}\left(i\right)\;+\;\beta_{1}\left(i\right)x_{i},\;for\;i\;=\;1\ldots n$$

where $Y_{i}$ is the estimated number of robberies, *x_i_* = the disaggregated population of the model, *β*_0_ and *β*_1_ are parameters that describe this relationship around a target zone i.

## 4. Results

### 4.1. Interpolation errors for Pop.CR1

**Figure 3.**
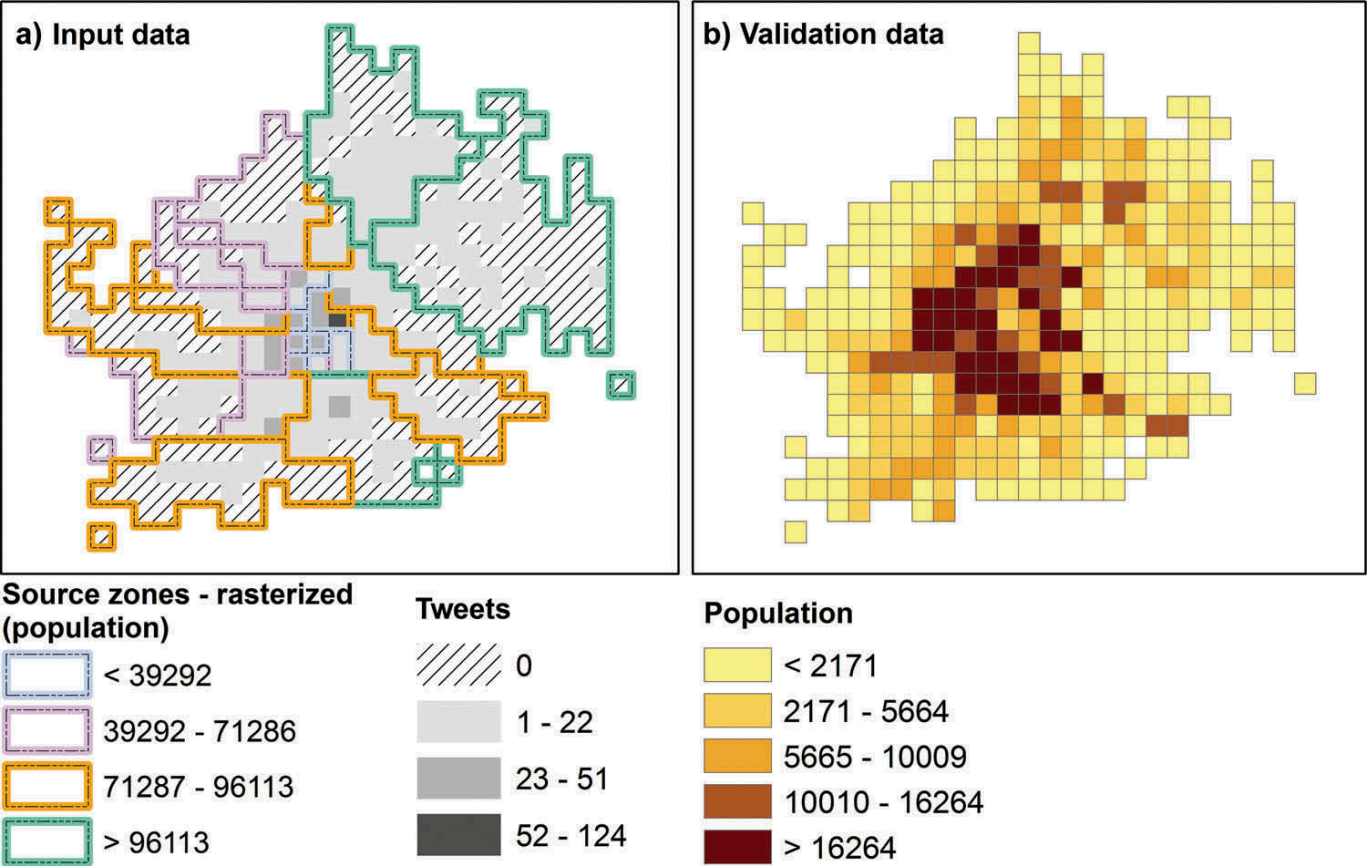
Input data: (a) rasterized source zones overlapping with counts of tweets per cell (tweets collected during workday sleeping hours), and (b) validation population data.

**Figure 4.**
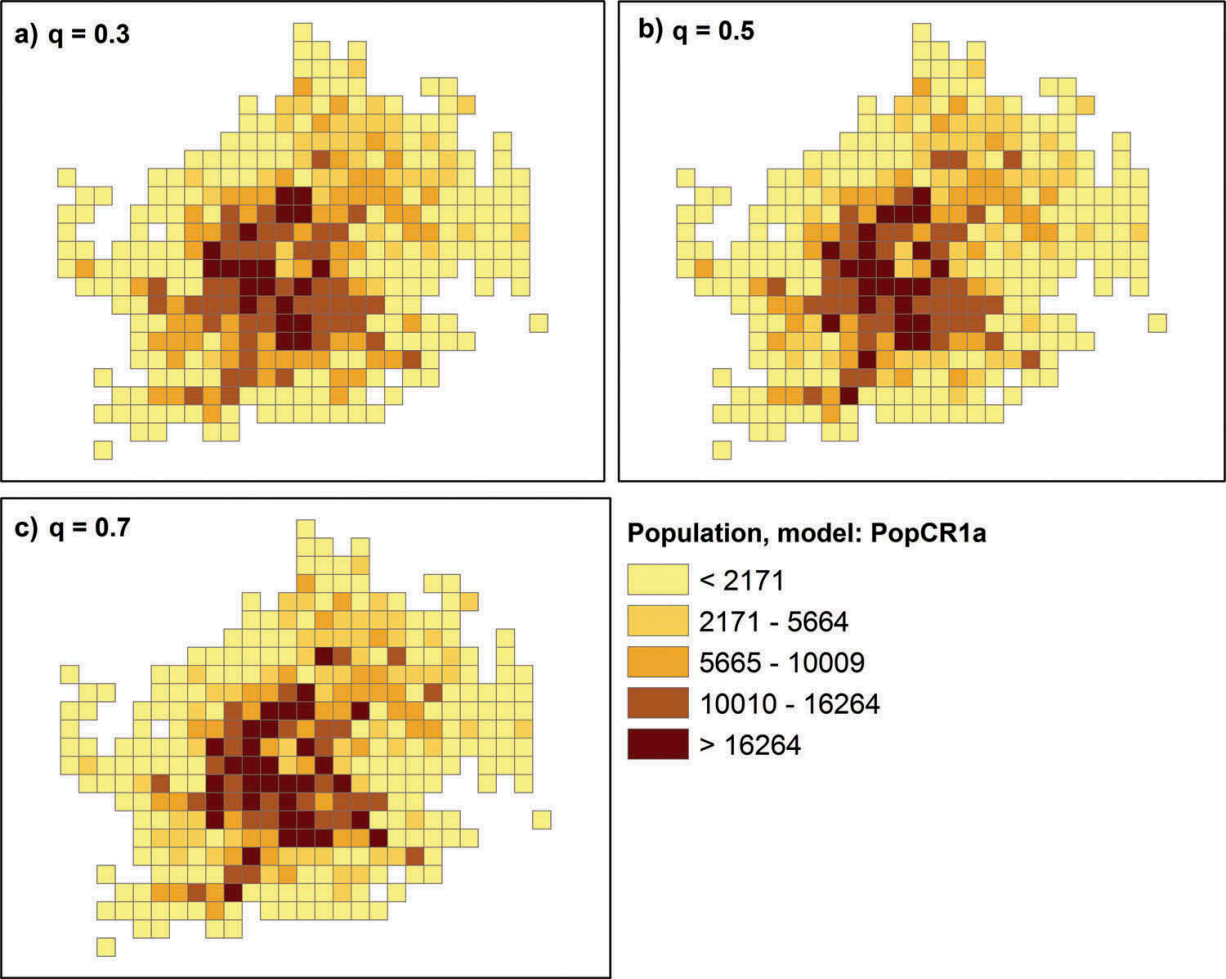
PopCR1a models: (a) power parameter q = 0.3, (b) q = 0.5, (c) q = 0.7.

The input data of the first model are shown in Figure 3 with three layers of information, namely the rasterized source zones classified by the population, counts of tweets per cell, and the distribution of the validation data. The first model, PopCR1a, was tested using several power parameters (i.e. “*q*”). By increasing *q*, the degree of local influence that the density of the tweets has on the model is increased as well. Thus, a smaller *q* in a model will deliver a smoother surface compared to a bigger one, as shown in Figure 4 – maps A, B, and C.

**Figure 5.**
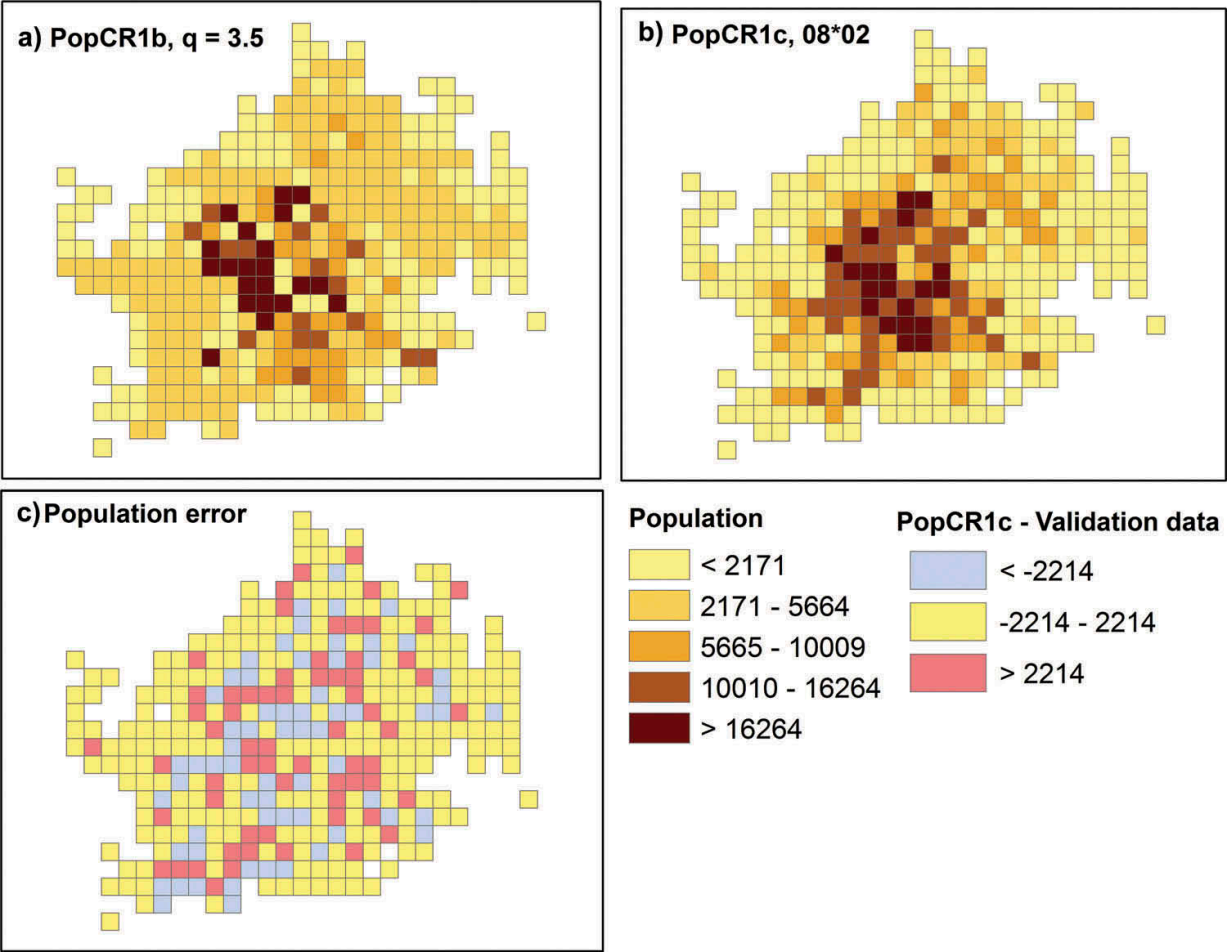
PopCR1b and 1c models: (a) best PopCR1b model, (b) best PopCR1c model, and (c) population difference between the PopCR1c and validation data.

Furthermore, we created a second model, PopCR1b, using an alternative set of control points. This is a dataset of schools’ locations (including nursery schools) in Vienna. The dataset was downloaded from the official platform for open governmental data in Austria (www.data.gv.at) and consists of 2588 locations. This type of control data was also used by Zhang and Qiu (2011) with the original distance function of the “point-based intelligent approach”. We similarly applied the distance function to the schools and tested several *q* parameters for this model. Then, we created a third model, PopCR1c, in which the final disaggregated value is influenced by both the disaggregated value using tweets (popCR1a) and using schools (popCR1b). In Table 2, results of the interpolation errors are presented for three of the tested parameters for each model, which yielded the best results, and also show a trend regarding the error. With respect to the first model based on tweets, the PopCR1a with a *q* = 0.5 gave the best results for three out of the four error measures. Generally, the interpolation error of a model increases as the absolute difference of the *q* value to 0.5 increases. The exception to this trend is the same *R* ^2^ value (i.e. 0.68) for both PopCR1a_q0.5 and PopCR1a_q0.7 models. For the second model, higher *q* values are associated with better results compared to the first model. In particular, values between 3.5 and 4.5 gave the best results in our application. *R* ^2^ and *r* values achieved the highest scores within this range. The RMSE is decreasing with decreasing *q* values up to *q* = 1, though the MAE has an opposite pattern up to a *q* = 4.5. Summarizing the error results of this model, we conclude that PopCR1b with a *q* = 3.5 is the best model based on schools. In general, all population models that use schools as control data (school-based models) underperform considerably the population models that use tweets as control data (tweets-based models). In the last mixed model, the disaggregated values of the PopCR1a_q0.5 and PopCR1b_q3.5 are used to calculate the final disaggregated value based on several weighting schemes (Table 2 shows again the best three results). The best results were achieved when PopCR1a_q0.5 weights 0.8 of the final cell value and PopCR1b_q3.5 weights 0.2 of the final cell value. This model, PopCR1c_08*02, minimized the RMSE and MAE and increased *R* ^2^ and *r* values, and thus is the best of all tested models. Models PopCR1b_q3.5 and PopCR1c_08*02 are shown in Figure 5 (maps A and B). Note that the school model, due to the distance function, creates a much smoother surface compared to the mixed model and the tweets model.

**Table 2.** Interpolation errors for all PopCR1 tested models of different power parameters (i.e. *q* = 0.3, 0.5, 0.7, 3.5, 4, 4.5, *and weighting scheme between* PopCR1a_q0.5 *and* PopCR1b_q3.5 *= *0.6 × 0.4, 0.8 × 0.2, 0.9 × 0.1).

Model	RMSE	MAE	*R*^2^	*r*
PopCR1a_q0.3	4001	2615	0.65	0.80
PopCR1a_q0.5	3945	2463	0.68	0.83
PopCR1a_q0.7	4253	2517	0.68	0.82
PopCR1b_q3.5	5412	3145	0.44	0.66
PopCR1b_q4	5509	3133	0.43	0.66
PopCR1b_q4.5	5612	3130	0.43	0.66
PopCR1c_06*04	3691	2172	0.68	0.83
PopCR1c_08*02	3655	2214	0.70	0.84
PopCR1c_09*01	3762	2304	0.69	0.83

Furthermore, in Figure 5 (map C), we visualized the population error (i.e. disaggregated population – validation population) for the best model, PopCR1c_08*02, to detect if there are any clearly defined areas for which the model does not perform well. The classification of the error shows cells with an error that is higher, lower, or within the – MAE to MAE. The map does not visualize clustered areas of a high overestimation or a high underestimation of the population. This was also tested with spatial autocorrelation statistics of the absolute population error in each cell. The results showed marginally dispersed and clustered patterns with values close to the theoretical ones (i.e. Getis–Ord General *G* = 0.03; *p* value: 0.001, Geary’s *C* = 1.03, *p* value: 0.01, Moran’s *I* = 0.04, *p* value: 0.001).

### 4.2. Spatial statistical results for Pop.CR2

**Figure 6.**
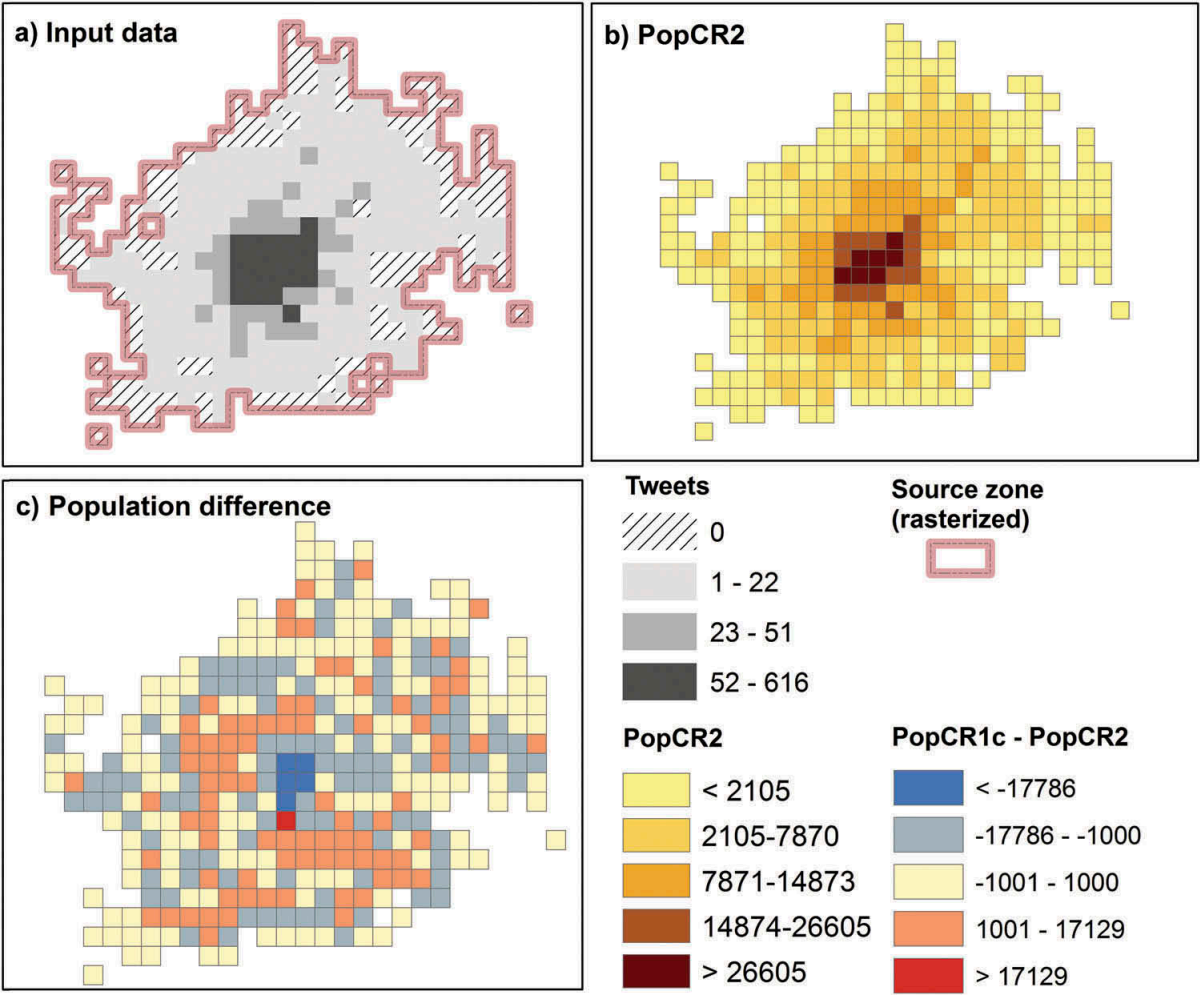
Input data and PopCR2 against PopCR1: (a) rasterized source zone overlapping with counts of tweets per cell (*tweets collected during weekend night hours*), (b) best PopCR2 model (*q* = 0.5), and (c) population difference between the PopCR1 and PopCR2.

A similar analytical approach as described above was employed for the second model. PopCR2_q0.5 (*q* = 0.5) and PopCR2_q0.7 (*q* = 0.7) gave the best spatial statistical results among several power parameters that were tested. Table 3 shows the Lr and the GWR results for the best four tested PopCR2 models and the comparison model, which is the best PopCR1 model (PopCR1c_08*02). PopCR2_q0.7 gives the highest mean Lr(0.69) and the vast majority of the cells (95%) are significantly positively correlated. PopCR2_q0.5 has the highest *R* ^2^ for the GWR (0.80) and has a mean absolute residual of 1.16. Generally, all models including the PopCR1 have positive local correlation coefficients in the majority of the cells and also high *R* ^2^ values. However, PopCR2 models have stronger relationships with robberies than the PopCR1 model. In Figure 6, the input data (i.e. source zone and tweets per cell), the best PopCR2 (*q* = 0.5), and the difference in population counts between PopCR1 and PopCR2 are shown in maps A, B, and C, respectively. Comparing maps B and C, we notice that the center of the city has higher population at weekend night times compared to workday sleeping hours.

**Table 3.** Results of the local correlation coefficients and geographically weighted regressions for four tested models and the comparison model (i.e. *PopCR1c*).

	Local correlationcoefficient		Geographicallyweighted regression	
Model	Percent of significantcorrelations^a^ (%)	Mean local correlationcoefficient	Mean absoluteresidual	*R*^2^
PopCR1c_08^a^02	84	0.61	1.29	0.75
PopCR2_q0.3	96	0.62	1.22	0.80
PopCR2_q0.5	96	0.67	1.16	0.80
PopCR2_q0.7	95	0.69	1.15	0.79
PopCR2_q1	92	0.68	1.21	0.76

^a^Correlation is significant at the 0.05 significance level.

**Figure 7.**
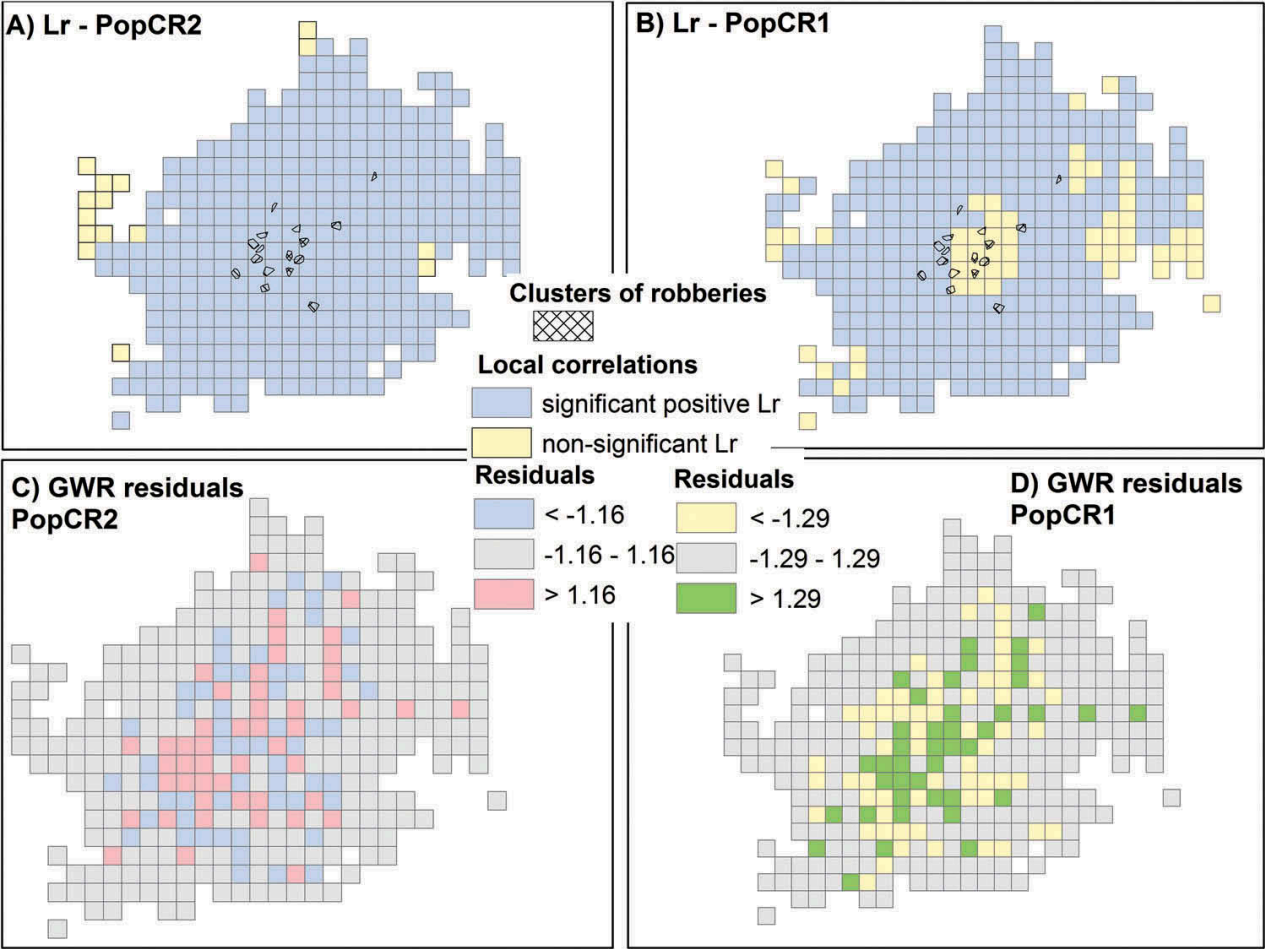
Statistical comparison of PopCR1 and PopCR2 (*best models*): (a) Significant local correlations of PopCR2 with robberies, (b) significant local correlations of PopCR1 with robberies, (c) geographically weighted regression residuals between PopCR2 and robberies, (d) geographically weighted regression residuals between PopCR1 and robberies.

The higher local correlation, Lr, of robberies with PopCR2 compared to robberies with PopCR1 is visually illustrated in Figure 7 (maps A and B). Although areas of robberies’ clusters are all positively correlated with disaggregated populations of PopCR2, a considerable amount of disaggregated populations located in the city center of PopCR1 is not significantly correlated with robberies’ clusters. Also, in maps C and D, we see the distribution of the GWR residuals that are higher, lower, or within the – MAR to MAR (i.e. mean absolute residual). The spatial autocorrelation of the absolute residuals in each cell was tested for both models to detect, if there are any clearly defined areas for which the underlying disaggregated population does not sufficiently estimate the number of robberies. Similar to the interpolation analysis, results showed nonsignificant or weak clustering and dispersed patterns. Results of the indices for PopCR1 are as follows: Getis–Ord General *G* = 0.05; *p* value: 0.001, Geary’s *C* = not significant, Moran’s *I* = 0.11; *p* value: 0.001. Results of the indices for PopCR2 are as follows: Getis-Ord General *G* = not significant, Geary’s *C* = 1.05; *p* value: 0.001, Moran’s *I* = 0.08; *p* value: 0.001.

## 5. Concluding remarks and future directions

This study developed and evaluated spatiotemporal models that depict the distribution of people prone to be victimized by burglaries and robberies. The models were created using a density-weighted interpolation that disaggregates the source data into smaller spatial units considering the spatial distribution of the ancillary data. Census data acted as source data for the existing population and Twitter data as ancillary data to redistribute the source data more accurately throughout the space. Although previous studies have used Twitter data to derive population at risk models for crime, this is the first attempt in which the temporal and the spatial attributes of the tweets were processed. The additional preprocessing of tweets allows creating distributions of a heightened crime risk that vary for each of the two crime types. The presented methodology can be used to derive population models as parameter estimates in crime prediction methods or as population information that can be used to calculate rates of offences more precisely.

To evaluate the models, two additional datasets were used: a gridded population dataset produced by Eurostat, and robberies of the study area in Vienna in 2011. The gridded population dataset was compared with the Pop.CR1 (i.e. the population model for burglaries) to assess the interpolation error. Several initial models were tested by changing the power parameter of the interpolation method and examining the results of the error measures. The best model was the one with a power parameter of 0.5. Also, additional models were created with: (a) a distance decay interpolation using schools as ancillary data and (b) a weighted interpolation between the interpolated values of the two sets of control points (i.e. schools and Twitter data). Tweets-based models outperformed all school-based models. However, the best model was achieved when the population value for each target zone was calculated as weighting function between the interpolated values of the best tweets-based model and the interpolated values of the best schools-based model at a ratio of 0.8–0.2, respectively. Generally, the interpolation error did not appear to be spatially concentrated.

Robberies were used to examine their spatial relationship with the Pop.CR2 (i.e. the population model for robberies). The analysis of the second model yielded similar results. The power parameters of 0.5 and 0.7 created models with the highest spatial correlation to robberies compared to models of smaller or larger power parameters. The analysis of residuals of the GWR showed that there were no concentrated areas where the disaggregated population did not estimate the number of robberies well. More importantly, Pop.CR2 predicted robberies better than Pop.CR1. This demonstrates that models tailored to the characteristic of a crime type has the potential to improve prediction results, which in turn raises new research challenges in population modeling and spatial crime analysis.

A major advantage of our method is the resolution at which models can be created, which can range from very fine to very coarse. This may be decided based on specific application purposes. The resolution of our models was set to 1 km^2^ to allow a direct comparison with the validation data. However, much smaller units such as street blocks or finer grid cells could have been used as well. The only precondition is that the model consists of areal units, thus deriving a point distribution is not possible. Other benefits are the method’s simplicity and that it relies on freely available Twitter data. However, we cannot claim that if it is used solely to disaggregate residential population, the interpolation results will be as accurate as other sophisticated interpolation methods that include a variety of ancillary data (e.g. LiDAR, OSM POIs, land use and land cover data) such as those that are referred to in Section 2. Furthermore, a limitation regarding the application of the method in this study is the incomplete source zone dataset that was used for creating Pop.CR2. The amount of mobile population in Vienna was considered equal to residents. This assumption does not capture tourists, people coming to the city for work or pleasure, and residents that leave the city during weekends. Hence, if Pop.CR2 is used for the calculation of robbery rates per grid cell, it may yield to an over or underestimation of actual values. Yet, these rates would be more accurate than rates that are calculated by the underlying residential population (i.e. Pop.CR1 model).

Upcoming research work will be dedicated to the application of this method to additional crime types against persons such as assaults, thefts, and pickpocketing. Furthermore, the textual information of tweets (i.e. the message itself and the profile information) can be exploited as additional filtering criteria of the population models. The testing of models should involve prediction methods such as the GWR that was used here, as well as the risk terrain method (Caplan, Kennedy, & Miller, 2011). Also, to further improve the calculation of offense rates and to create more accurate source zone datasets, special attention should be given to identify additional information on population flows within a city (e.g. workers and tourists) and to incorporate this information with the census population. Apart from the crime analytical purposes, our approach is replicable for population at risk studies. In the field of health geography, the interpolation technique can be used to produce models that depict exposure to diseases at fine resolutions when detailed population data are not available. Last, high-resolution spatiotemporal population models can complement or be combined with recent methods of modeling ambient population in disaster management studies (Khakpour & Rød, 2013; Smith, Martin, & Cockings, 2016).
